# Influence of Combined Transcranial Direct Current Stimulation and Motor Training on Corticospinal Excitability in Children With Unilateral Cerebral Palsy

**DOI:** 10.3389/fnhum.2019.00137

**Published:** 2019-04-24

**Authors:** Samuel T. Nemanich, Tonya L. Rich, Chao-Ying Chen, Jeremiah Menk, Kyle Rudser, Mo Chen, Gregg Meekins, Bernadette T. Gillick

**Affiliations:** ^1^Divisions of Physical Therapy and Rehabilitation Science, Department of Rehabilitation Medicine, University of Minnesota, Minneapolis, MN, United States; ^2^Department of Rehabilitation Sciences, The Hong Kong Polytechnic University, Kowloon, Hong Kong; ^3^Clinical and Translational Science Institute, Biostatistics, Design, and Analysis Center, University of Minnesota, Minneapolis, MN, United States; ^4^School of Public Health, Division of Biostatistics, University of Minnesota, Minneapolis, MN, United States; ^5^Non-invasive Neuromodulation Laboratory, University of Minnesota, Minneapolis, MN, United States; ^6^Department of Neurology, University of Minnesota, Minneapolis, MN, United States

**Keywords:** transcranial direct current stimulation, cerebral palsy, pediatric hemiparesis, corticospinal excitability, rehabilitation

## Abstract

Combined non-invasive brain stimulation (NIBS) and rehabilitation interventions have the potential to improve function in children with unilateral cerebral palsy (UCP), however their effects on developing brain function are not well understood. In a proof-of-principle study, we used single-pulse transcranial magnetic stimulation (TMS) to measure changes in corticospinal excitability and relationships to motor performance following a randomized controlled trial consisting of 10 days of combined constraint-induced movement therapy (CIMT) and cathodal transcranial direct current stimulation (tDCS) applied to the contralesional motor cortex. Twenty children and young adults (mean age = 12 years, 9 months, range = 7 years, 7 months, 21 years, 7 months) with UCP participated. TMS testing was performed before, after, and 6 months after the intervention to measure motor evoked potential (MEP) amplitude and cortical silent period (CSP) duration. The association between neurophysiologic and motor outcomes and differences in excitability between hemispheres were examined. Contralesional MEP amplitude decreased as hypothesized in five of five participants receiving active tDCS immediately after and 6 months after the intervention, however no statistically significant differences between intervention groups were noted for MEP amplitude [mean difference = −323.9 μV, 95% CI = (−989, 341), *p* = 0.34] or CSP duration [mean difference = 3.9 ms, 95% CI = (−7.7, 15.5), *p* = 0.51]. Changes in corticospinal excitability were not statistically associated with improvements in hand function after the intervention. Across all participants, MEP amplitudes measured in the more-affected hand from both contralesional (mean difference = −474.5 μV) and ipsilesional hemispheres (−624.5 μV) were smaller compared to the less-affected hand. Assessing neurophysiologic changes after tDCS in children with UCP provides an understanding of long-term effects on brain excitability to help determine its potential as a therapeutic intervention. Additional investigation into the neurophysiologic effects of tDCS in larger samples of children with UCP are needed to confirm these findings.

## Introduction

Non-invasive brain stimulation (NIBS), such as transcranial direct current stimulation (tDCS), can be used to modulate corticospinal excitability and produce changes in motor function (Peters et al., 2016). In people with stroke, ipsilesional corticospinal excitability is often lower than contralesional excitability (McDonnell and Stinear, 2017), due to both decreased neural activity in the ipsilesional hemisphere as well as excessive interhemispheric inhibition (IHI) from the contralesional hemisphere (Murase et al., 2004; Duque et al., 2005). NIBS interventions such as tDCS aim to enhance or reduce brain excitability depending on electrode polarity. Although the mechanism of action of tDCS is not fully understood, anodal tDCS increases neural excitability while cathodal tDCS decreases excitability (Nitsche et al., 2008). Currently, there is evidence supporting anodal and cathodal tDCS, when paired with other motor training interventions, in adult stroke. Anodal tDCS applied to the ipsilesional hemisphere directly targets the damaged cortex to restore neural excitability and aid in functional recovery (O’Shea et al., 2014; Allman et al., 2016). Alternatively, cathodal contralesional tDCS is hypothesized to reduce exaggerated IHI, potentially increasing excitability in the ipsilesional hemisphere following stroke. This hypothesis is supported by existing studies in adults with stroke demonstrating that cathodal contralesional tDCS augments the effects of intensive motor training compared to training alone (Bolognini et al., 2011; Nair et al., 2011; Figlewski et al., 2017).

Children with unilateral cerebral palsy (UCP), who experience a stroke or brain injury around the time of birth, demonstrate atypical patterns of corticospinal tract (CST) development and organization (Berweck et al., 2008; Staudt, 2010). which contributes to an imbalance in excitability between the lesioned and non-lesioned hemispheres (Berweck et al., 2008; Chen et al., 2016). These neural changes underlie the limitations in upper-extremity function (Holmström et al., 2010) that impact independence throughout their lifetime. Therefore, children with UCP may likewise benefit from innovative technologies incorporating NIBS and motor training to improve long-term function.

Combined tDCS and intensive motor training interventions have recently been investigated in children with UCP, with results showing some positive changes in neurophysiologic responses, motor function, and activities of daily living skills (Grecco et al., 2014; Collange Grecco et al., 2015; Kirton et al., 2017; Gillick et al., 2018b; Rich et al., 2018). While these initial studies have focused on establishing the safety of these combined tDCS and rehabilitation interventions and their effects on behavioral, little is known regarding their on influence corticospinal excitability in children with UCP. An understanding of how the addition of tDCS, in combination with a behavioral interventions, impacts brain function and on-going development after early brain injury is critical in evaluating the efficacy of these interventions as potential therapies for children with UCP.

Assessments of corticospinal excitability and connectivity using transcranial magnetic stimulation (TMS) have been found to be safe and feasible in pediatric populations (Krishnan et al., 2015; Allen et al., 2017). While motor evoked potential (MEP) amplitude is the most commonly studied measure of CST excitability, cortical silent periods (CSPs) may be a complementary measurement of inhibitory influence obtained with single-pulse TMS testing (McDonnell and Stinear, 2017). CSP is the duration of inhibition during voluntary muscle contraction with contributions from both spinal and supraspinal inhibitory inputs, and may reflect GABA_B_ activity in these areas (Cantello et al., 1992; McDonnell et al., 2006). Prior studies have examined these measures of corticospinal excitability, both before and after interventions to begin to identify neurophysiology mechanisms in children with UCP (Chen et al., 2016; Zewdie et al., 2017; Kuo et al., 2018; Rich et al., 2018). Specifically, Kuo et al. (2018) describe intervention-induced changes in contralesional corticospinal excitability following intensive therapy and repetitive TMS, and their relationship to motor function outcomes. Unique to tDCS, some decreases in contralesional corticospinal excitability were found in an open-label study of combining cathodal tDCS and bimanual therapy (Rich et al., 2018). Although promising, the current paucity of available data of the effects of combined tDCS and motor training interventions on brain excitability reveal gaps in our understanding of how tDCS modulates the developing brain in children with UCP.

To advance the application of combined neuromodulatory and motor training interventions in children with UCP, we examined corticospinal excitability before and after a randomized, sham-controlled clinical trial consisting of combined tDCS concurrent with a behavioral intervention. Our goal was to address gaps in knowledge related to individualized tDCS applications related to targeting TMS-derived motor hotspots, long-term assessment of excitability, and appropriate neurophysiologic biomarkers to guide future interventions. We predicted that contralesional corticospinal excitability would decrease following serial sessions of cathodal contralesional tDCS and ipsilesional excitability would increase based on the theory of rebalancing IHI as demonstrated in other studies involving repetitive TMS to modulate the non-lesioned cortex (Kirton et al., 2010; Cassidy et al., 2015). Furthermore, we compared the excitability of ipsilesional and contralesional responses across time to investigate the excitability of each corticospinal system. While both anodal ipsilesional and cathodal contralesional tDCS montages have been explored in previous studies of adult stroke, insufficient evidence exists in favor of one specific montage. For this study, we used a cathodal contralesional montage to ensure there was an identifiable cortical target where we can place the tDCS electrode, as well as to avoid the effects of the lesion on distribution of the electric field produced by tDCS (Minjoli et al., 2017). Overall, we hope this proof-of-principle investigation will offer insight into the optimal dosing parameters and potential mechanisms of action of combined neuromodulatory and behavioral interventions in children with UCP.

## Materials and Methods

### Participants

Twenty children with UCP participated in this clinical trial originally designed to assess the safety, feasibility, and efficacy of combined tDCS and constraint-induced movement therapy (CIMT) to improve hand function (Gillick et al., 2018b). Participants were included if they met the following criteria: (1) aged 7–21 years; (2) imaging-confirmed diagnosis of hemispheric stroke or periventricular leukomalacia (PVL); and (3) ability to follow 2-step commands. Participants were excluded if they met any of these criteria: (1) other neurological diagnosis; (2) history of seizure in the past 2 years; (3) history of injections for spasticity management within the last 6 months; (4) indwelling metal or devices and (5) absence of contralesional hemisphere MEP. Before enrolling, participants under the age of 18 provided assent and both caregivers provided written consent; participants over the age of 18 provided written consent. This study was approved by the Institutional Review Board at the University of Minnesota.

### Study Design

This study was part of a previously published randomized controlled trial (NCT02250092) designed to detect differences in hand function as a result of the intervention (Gillick et al., 2018b) Participants were evaluated within 11 days prior to (Pre-test, average 5 days between Pre-test and intervention), within 5 days after (Post-test, average 2 days between intervention and Post-test), and 6-months after Follow-up average 173 (days between intervention and Follow-up) the intervention. After enrolling, participants were stratified based on the presence or absence of a MEP in the contralateral hand following stimulation of the ipsilesional hemisphere, and then randomly assigned to sham (Sham+CIMT) or active (Active+CIMT) intervention group. Participants, caregivers, and study personnel involved with the behavioral intervention and assessments were blinded to group assignment. Because the primary analysis of behavioral results was previously published (Gillick et al., 2018b), MEP amplitude and CSP duration were analyzed after the intervention group assignments were revealed to study personnel.

### Interventions

The intervention consisted of 10 consecutive weekdays of combined tDCS and CIMT. tDCS (1 × 1 LTE, Soterix Medical, New York, NY, USA) was applied for 20 min with the cathode positioned over the TMS-derived motor hotspot of the contralesional hemisphere, and the anode positioned over the contralateral forehead. The Active+CIMT group received 0.7 mA stimulation, an intensity based on previous modeling of intracranial electric fields in pediatric UCP and safety data from a single-tDCS session study (Gillick et al., 2014). For the Sham+CIMT group, the tDCS unit was set to a built-in sham setting, which extinguished the current after a 30-s to 1-min ramp-up phase. The current then was gradually re-introduced during a ramp-down phase during the last 60 s of the 20-min session. Each participant received CIMT concurrently with tDCS and an additional 100 min without tDCS (total 120 min of CIMT). The tDCS+CIMT intervention occurred in small groups of up to three children paired one-to-one with trained interventionists. For CIMT, the less-affected upper limb was placed in a sling and each child was engaged in activities for motor skill development of the more-affected hand. As little is known about how tDCS directly affects motor function in real time, the activities were designed to relate to the child’s goals and align with current clinical practice (e.g., fine motor, activities of daily living skills and leisure activities). A trained interventionist was paired with each participant to engage the child in shaping activities. As shown in previous clinical trials, this form of therapy can improve unimanual function in children with UCP (Charles et al., 2006; Gordon et al., 2008).

### Assessments

#### Behavioral

We assessed hand function using the Assisting Hand Assessment (AHA), a valid and reliable tool measuring bimanual function during a functional task (Krumlinde-Sundholm et al., 2007), as a one of several motor assessments (Gillick et al., 2018b). The AHA was administered by a blinded and AHA-certified therapist and scored by separate blinded AHA-certified therapist. The raw AHA score was converted to a logit scale for analysis (Krumlinde-Sundholm, 2012).

#### Safety

During TMS testing and tDCS interventions, safety and tolerance were monitored and documented using a participant report of symptoms checklist modified from Garvey et al. (2001a) and Gillick et al. (2015b). Any adverse events noted or reported by the participant were reviewed by investigators and caregivers to determine the relation to study procedures and were reported for review by the study medical monitor.

#### Transcranial Magnetic Stimulation

Corticospinal excitability of both hemispheres was assessed using single-pulse TMS (Bistim; McDonnell and Stinear, 2017, The Magstim Co., Dyfed, UK). Participants were seated in a comfortable chair and were instructed to inform the researchers of any discomfort during the testing session. TMS pulses were delivered using a 70 mm, figure-of-eight coil (Magstim) placed tangentially on the scalp with the handle positioned 45 degrees posterior-lateral to the longitudinal fissure. Coil position was superimposed on a three-dimensional brain model obtained from individual’s T1-weighted anatomical images from previously-attained MRIs and guided using a stereotactic neuronavigation system (Brainsight, Rogue Research, Montreal, QC, Canada). Stainless steel surface electromyography (EMG) electrodes were placed bilaterally on the first dorsal interosseous (FDI) to record muscle responses to stimulation. EMG data were acquired and displayed on a custom-built system using LabView software (National Instruments, Austin, TX, USA).

#### Motor Hotspot Identification and Resting Motor Threshold Determination

At Pre-test, evaluation of the motor hotspot location began at 50% maximum stimulator output (MSO) and commenced at the hand knob region of the contralesional primary motor cortex (M1) as previously described (Rich et al., 2017). This location was approximated based on each child’s T1-weighted magnetic resonance imaging (MRI). MSO was increased by increments of 5% until MEPs of at least 50 μV in amplitude were observed from the contralateral hand in three of five trials. Then, MSO was decreased by 1% for that location to determine the resting motor threshold (RMT), defined as the lowest intensity that evoked a MEP greater than 50 μV in three out of five consecutive trials. The coil was then systematically relocated to alternative locations (1 cm anterior, posterior, lateral and medial to the original hotspot location) to determine a new potential hotspot. The final hotspot was used for TMS testing at Pre-test, Post-test and Follow-up assessments. This procedure was repeated for the ipsilesional hemisphere. Stimulation intensity was limited to 85% MSO for all testing as established in our approved TMS protocol to ensure comfort and tolerability (Gillick et al., 2015c). Therefore, participants with RMTs above 71% MSO, which would require a suprathreshold testing intensity greater than 85% MSO, did not undergo excitability testing beyond motor hotspot localization. These exclusions (*n* = 2) were limited to testing the ipsilesional hemispheres (Supplementary Table S1).

#### Motor Evoked Potential Amplitude and Cortical Silent Period Duration

Following motor hotspot identification, MEP amplitudes and CSP duration measures were assessed. To measure cortical excitability, we measured MEP amplitudes in both FDIs during stimulation of the contralesional hemisphere. With the coil positioned over the M1 hotspot and the muscle at rest (± 10 μV), 10 consecutive single TMS pulses were delivered at 120% Pre-test RMT and a frequency of 0.1 Hz.

We then assessed the CSP duration to determine the potential impact of cathodal tDCS on inhibitory circuits. For CSP testing, we first determined the maximum voluntary contraction (MVC) of the contralateral FDI using an isometric contraction. Participants who lacked selective motor control for individual finger muscles grasped small objects (e.g., foam balls) to elicit FDI activity. After MVC was established, participants were asked to maintain a contraction at 20% of MVC using visual feedback from a computer display and verbal cueing from researchers. Ten CSP trials were collected at an intensity of 120% Pre-test RMT with the coil positioned over the M1 hotspot. Rest breaks of at least 10 s were provided between trials to minimize fatigue. These procedures were repeated for the ipsilesional hemisphere if an ipsilesional motor hotspot was present.

### Data and Statistical Analysis

Raw EMG data were downloaded and digitally filtered using a band-pass filter (10–2,000 Hz). Filtered data were rectified and smoothed using a 10 ms moving standard deviation window. MEP amplitude was defined as the peak-to-peak amplitude of the filtered unrectified EMG signal, and the average amplitude of collected trials is reported. Trials with no measurable MEP, or high pre-stimulus EMG activity (exceeded 20 μV peak-to-peak), were excluded from analysis (<5%). For analysis of CSP duration, we identified the MEP offset and return of EMG activity semi-automatically using custom Matlab scripts (The Mathworks, Natick, MA, USA) employing the method from Garvey et al. (2001b). MEP offset was defined as the time where the smoothed EMG signal fell below pre-stimulus EMG activity for 10 consecutive ms. Upper and lower deviation limits were calculated based on the mean consecutive difference of rectified pre-stimulus EMG activity. Return of EMG activity following stimulation was defined as the time where 50% of the rectified EMG signal surpassed the lower deviation limit in a 5 ms window. CSP duration was calculated as the time difference between the return of EMG activity and MEP offset (Supplementary Table S2). Each trial was visually assessed for accuracy of the semi-automatic program. Fewer than 15% of trials required manual determination of CSP duration, primarily due to incorrect determination of the return of EMG activity.

Mean and standard deviations were reported for individual and group neurophysiologic data. We used general linear models with a Gaussian link to compare the change in the primary neurophysiologic outcomes, contralesional M1 MEP amplitude and CSP duration, between Active+CIMT and Sham+CIMT groups following the intervention, adjusting for Pre-Test values which in some cases were different between groups (Senn, 2006). Robust standard errors were used for confidence intervals and *P*-values. Secondary analyses of MEP amplitude between hemispheres combined the multiple trials at a given time point into a single measurement per person for each of Pre-test, Post-test, and Follow-up. Differences were evaluated using generalized estimating equations with an exchangeable working correlation structure to account for the correlation between multiple measurements on the same participants and robust variance estimation for confidence intervals and *P*-values. Finally, the relationship between neurophysiologic variables and the AHA was analyzed using linear regression, adjusting for age. Analysis was performed using (R Core Team, 2016) and Matlab, and a significance level of *p* < 0.05 was used for all statistical tests. All analyses were performed *post hoc* as this study was powered to detect changes in clinical hand function (AHA; Gillick et al., 2015c).

## Results

### Demographics/Participants Overview

Table 1 summarizes the participant characteristics for each group and Pre-test neurophysiologic measures. Age, sex, MACS, side of hemiparesis, and Pre-test AHA were comparable between groups. One participant presented with bilateral, asymmetric PVL, while the remaining 19 participants had a cortical and/or subcortical lesions. Of the neurophysiologic measures, only contralesional M1 MEP amplitude was significantly greater in the Active+CIMT group at Pre-test (*p* = 0.04). No MEP was identified in the ipsilesional hemisphere of 8 of 20 participants. No serious adverse events occurred in any testing or intervention session. A summary of the transient minor adverse events was summarized in a prior publication (Gillick et al., 2018a).

**Table 1:** Participant characteristics.

Characteristic	Sham+CIMT (*n* = 10)	Active+CIMT (*n* = 10)
Age (min, max)	13 y 2 m (8 y 2 m, 21 y 7 m)	12 y 4 m (7 y 7 m, 16 y 11 m)
Sex (male/female)	4/6	5/5
MACS	I (1), II (8), IV (1)	I (1), II (8), III (1)
Side of hemiparesis	8 right, 2 left	7 right, 3 left
Ipsilesional MEP present	6 Y, 4 N	6 Y, 4 N
Contralesional RMT (% MSO)	54.8 ± 14.1	55.4 ± 11.4
Ipsilesional RMT (% MSO)	62.6 ± 7.82	65.7 ± 12.2
Pre-Test Contralesional M1 MEP amplitude (μV)	320 ± 298	769.9 ± 261*
Pre-Test Contralesional M1 CSP Duration (ms)	113 ± 39.5	101 ± 45.6

Continuous variables are presented as mean ± SD; MACS, Manual Ability Classification Scale; CIMT, constraint-induced movement therapy; RMT, resting motor threshold; IL, ipsilesional; CL, contralesional. MEP, motor evoked potential; CSP, cortical silent period; *significantly different than Sham+CIMT, *p* < 0.05.

#### Determination of Included Data

**Figure 1:**
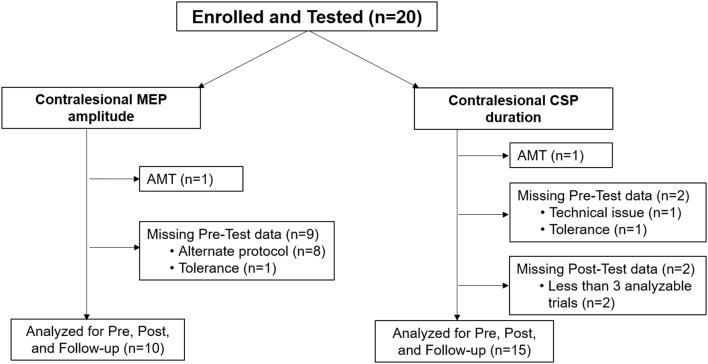
Participant flow diagram indicating the number of children assessed for analysis of contralesional motor evoked potential (MEP) amplitude and analysis of cortical silent period (CSP) duration at Pre-test, Post-test, and Follow-up periods. Participants were excluded from analysis primarily if there were missing Pre-test data. AMT, active motor threshold.

Data were collected within the participant’s tolerance and neurophysiologic characteristics (i.e., active motor threshold-AMT, inconclusive EMG responses). We excluded data from the primary analysis of contralesional M1 MEP amplitude and CSP duration for the reasons of active motor threshold and missing Pre-test data (Figure 1). Ipsilesional M1 MEP amplitude and CSP duration results were further limited by lack of MEPs and high RMTs in participants with measurable ipsilesional M1 MEPs.

### Contralesional M1 Testing

#### Contralesional M1 MEP Amplitude

**Figure 2:**
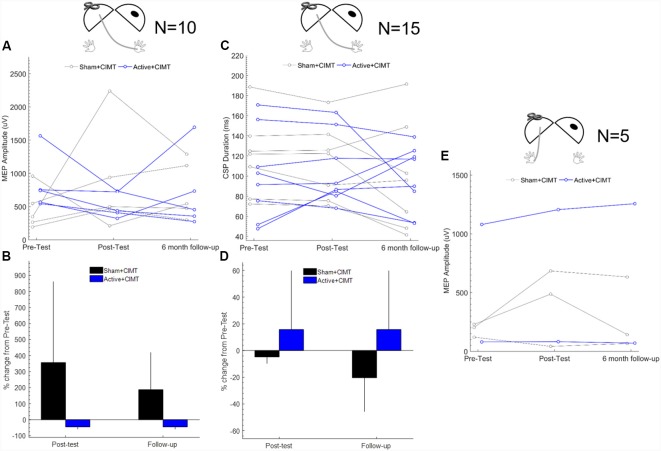
Contralesional M1 corticospinal excitability following transcranial direct current stimulation (tDCS)+constraint-induced movement therapy (CIMT). **(A)** Individual mean MEP amplitude measured in less-affected FDI across all assessment times (*n* = 10). **(B)** Group mean percent change in MEP amplitude measured from Pre-test; error bars are 1 SD (*n* = 10). **(C)** Individual CSP duration measured in less-affected FDI across all assessment times (*n* = 15). **(D)** Group mean percent change in CSP duration from Pre-test; error bars are 1 SD (*n* = 15). **(E)** Individual MEP amplitude measured in more-affected FDI across all assessment times (*n* = 5). Diagrams above each plot show the location of stimulation and recording, where the dark oval represents the damaged hemisphere. FDI, first dorsal interosseous; MEP, motor evoked potential.

Individual MEP amplitude in the less-affected (i.e., stronger) FDI (*n* = 10) obtained from contralesional M1 testing are shown in Figure 2A. MEP amplitude decreased in the Active+CIMT group (−45.2 ± 14.2%) and increased in the Sham+CIMT group (356 ± 505%) from Pre-test to Post-test (Figure 2B). Furthermore, 5/5 children in the Active+CIMT group exhibited smaller MEP amplitudes at Post-test compared to Pre-test, compared to 1/5 in the Sham+CIMT group. Adjusting for Pre-test MEP amplitude, the differences comparing the Active+CIMT group vs. the Sham+CIMT group at Pre-test and Post-test [mean difference = −323.9 μV, 95% CI = (−989, 341), *p* = 0.34] and from Pre-test to Follow-up [mean difference = −17.2 μV, 95% CI = (−630, 596), *p* = 0.96] were not statistically significant. MEPs in the more-affected (i.e., weaker) FDI from stimulation of contralesional M1 were detected in 5/10 participants at all time points (Figure 2E). Analysis of the effects of the tDCS/CIMT intervention on these responses was not performed due to the small sample.

#### Contralesional M1 CSP Duration

Individual CSP duration (*n* = 15) measured during contralesional M1 testing are shown in Figure 2C. The average change from Pre-test to Post-test in contralesional CSP duration was 15.7 ± 44.0% in the Active+CIMT group and −4.80 ± 4.91% in the Sham+CIMT group (Figure 2D). On an individual level, 3/8 participants in the Active+CIMT showed an increased in CSP duration, compared to 0/7 in the Sham+CIMT group. After adjusting for Pre-test CSP duration, no significant difference was found when comparing contralesional CSP duration between the Active+CIMT vs. Sham+CIMT groups at Post-test [mean difference = 3.9 ms, 95% CI = (−7.7, 15.5), *p* = 0.51]. Similarly, no significant group difference was found at Follow-up [mean difference = 20.6 ms, 95% CI = (−1.5, 42.7), *p* = 0.07].

### Ipsilesional M1 Testing

#### Ipsilesional M1 MEP Amplitude

**Figure 3:**
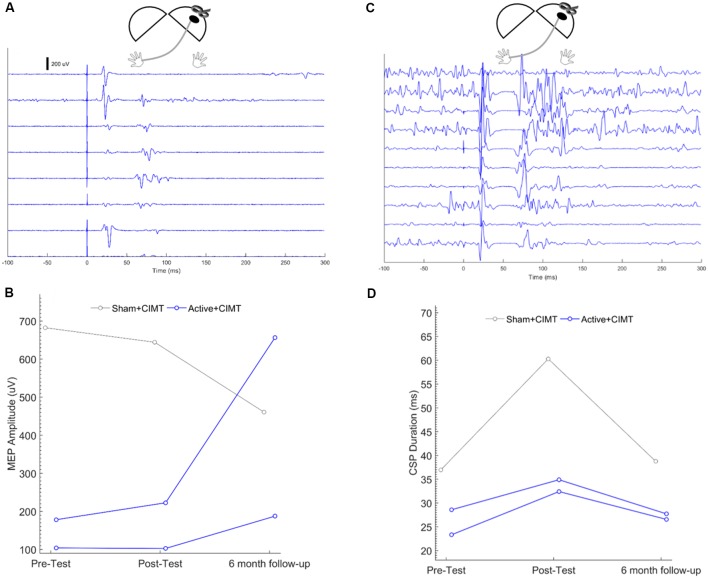
Ipsilesional M1 corticospinal excitability following tDCS+CIMT. **(A)** Representative traces from seven trials of single-pulse transcranial magnetic stimulation (TMS) testing of ipsilesional M1 in a single participant. Each trace represents unrectified electromyography (EMG) activity, where Time = 0 represents the onset of the TMS pulse. **(B)** Individual mean MEP amplitude measured across all participants in more-affected FDI across all assessment times (*n* = 3). **(C)** Representative traces from 10 trials of CSP testing of ipsilesional M1 in a single participant. Each trace represents unrectified EMG activity, where Time = 0 represents the onset of the TMS pulse. **(D)** Individual CSP duration measured across all participants in more-affected FDI across all assessment times (*n* = 3). Diagrams above each plot show the location of stimulation and recording, where the dark oval represents the damaged hemisphere. FDI, first dorsal interosseous; MEP, motor evoked potential.

Representative ipsilesional MEP traces measured at Pre-test from one participant are shown in the waterfall plot in Figure 3A. MEP amplitude was measured from the ipsilesional M1 in three participants at all time-points (Figure 3B). The mean difference in ipsilesional MEP amplitude from Pre-test to Post-test was 11.8 ± 18.6% for Active+CIMT (*N* = 2) and was −5.59% for Sham+CIMT (*N* = 1). The mean difference in ipsilesional MEP amplitude from Pre-test to Follow-up was 174 ± 133% for Active+CIMT and was −32.5% for Sham+CIMT. Additional statistical analyses of the effects of the tDCS/CIMT intervention on ipsilesional MEP amplitude were not performed due to the small sample and missing observations.

#### Ipsilesional M1 CSP Duration

Representative CSP traces from ipsilesional CSP testing from one participant, are shown in the waterfall plot in Figure 3C. CSPs were measured from the ipsilesional M1 in three participants at all time points (Figure 3D; see “Cathodal Contralesional tDCS and Exaggerated IHI” section for discussion of ipsilesional hemisphere CSP measurement). The mean difference in ipsilesional MEP amplitude from Pre-test to Post-test was 30.5 ± 11.9% for Active+CIMT (*N* = 2) and was 63.2% for Sham+CIMT (*N* = 1). The mean difference in ipsilesional MEP amplitude from Pre-test to Follow-up was 5.42 ± 11.9% for Active+CIMT and was 4.87% for Sham+CIMT. Additional statistical analyses of the effects of the tDCS/CIMT intervention on ipsilesional CSP duration were not performed due to the small sample and missing observations.

### Comparison of Contralesional and Ipsilesional M1 MEPs

**Figure 4:**
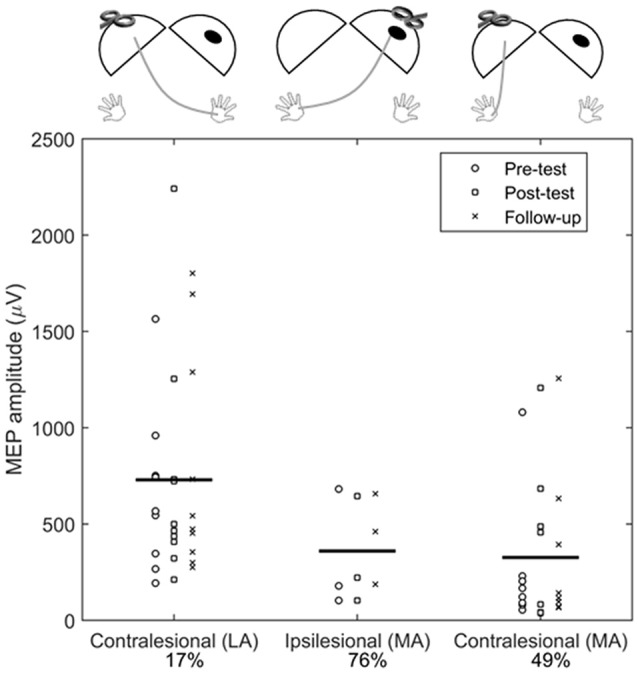
Comparison of contralesional and ipsilesional M1 MEP amplitudes. Each symbol represents the average of an individual participant at Pre-test (°), Post-test (□), and Follow-up (X) assessments. Horizontal bar is the grand mean across all assessments. The percentages indicated the proportion of absent MEP responses during testing (e.g., for ipsilesional M1 testing, 76% of trials did not elicit an MEP). Diagrams above each plot show the location of stimulation and recording, where the dark oval represents the damaged hemisphere. LA, less-affected FDI; MA, more-affected FDI.

Across all assessments, MEPs in the less-affected FDI (from contralesional M1 testing) were larger in amplitude compared to MEPs in the more-affected FDI when testing the ipsilesional (mean difference = −624.5 μV, 95% CI: −896.5, −352.6 μV, *p* < 0.001) and contralesional (mean difference = −474.5 μV, 95% CI: −76.4, −185.5 μV, *p* = 0.001) M1 (Figure 4).

### Correlation of Neurophysiology and Motor Outcomes

While all participants showed statistically significant improvements in hand function as measured by the AHA, there were no statistically significant differences on the AHA between the Active+CIMT and Sham+CIMT groups as were previously reported in a separate publication (Gillick et al., 2018b). Partial correlation analysis showed that neither Pre-test contralesional M1 MEP amplitude (*r* = 0.39, *p* = 0.29) or CSP duration (*r* = 0.09, *p* = 0.75) was significantly related to Pre-test AHA score, after adjusting for age. Furthermore, there was no statistically significant relationship between the change in contralesional M1 MEP amplitude (*r* = 0.36, *p* = 0.34) and CSP duration (*r* = −0.13, *p* = 0.62) when compared to change in AHA from Pre-test to Post-test.

## Discussion

In this study, we compared the changes in corticospinal excitability as measured by single-pulse TMS in children with UCP after serial sessions of active or sham tDCS paired with CIMT. Although not statistically significant, after a cathodal contralesional tDCS and CIMT intervention, we observed reductions in contralesional M1 MEP amplitude in the all (5 of 5) participants in the Active+CIMT compared to 1 of 5 participants in the Sham+CIMT group, whereas no significant change was noted at the individual or group level for contralesional CSP duration. Furthermore, across all participants and assessment times, MEP amplitudes measured in the more-affected FDI were smaller compared to the less-affected FDI. Although the data in our study were collected as part of a larger clinical trial powered to detect changes in clinical hand function outcomes and not neurophysiologic responses, we were able to detect meaningful differences in corticospinal excitability at the individual level, as well as differences in excitability when comparing responses from each hemisphere. We now discuss the implications of these findings in the context of neuromodulatory interventions and corticospinal excitability testing in children with UCP.

### Effect of tDCS/CIMT on M1 Excitability

There are many potential neurophysiologic measures obtained from single-pulse TMS assessments that can indicate changes in brain function following neuromodulatory interventions (Kirton, 2017). MEP amplitude is a measure of corticospinal excitability that has consistently shown to be a useful neurophysiologic biomarker of change following tDCS in adult studies (Horvath et al., 2015). Recently, Kuo et al. (2018) examined changes following combined motor training and repetitive TMS, finding an overall increase in contralesional MEP amplitude. However, the specific effects of tDCS on M1 excitability have been less examined in children with UCP.

By using an inhibitory form of tDCS, we anticipated a decrease in contralesional M1 and an increase in ipsilesional M1 excitability *via* disinhibition of exaggerated IHI. Although not significant at the group level, our results of decreased MEP amplitude found in all participants in the Active+CIMT group provide support of a potential inhibitory effect of cathodal tDCS on contralesional excitability. Analysis of excitability measures in the ipsilesional M1 showed that MEP amplitude was significantly smaller across all measured MEPs as compared to the contralesional M1. These findings are in agreement with previous results in adult stroke (McDonnell and Stinear, 2017) and UCP (Berweck et al., 2008; Vry et al., 2008; Mackey et al., 2014) studies which showed reduced ipsilesional corticospinal excitability, reflecting the imbalance in activity across hemispheres. However, the small sample of ipsilesional M1 MEP amplitude data reported in our study limit any conclusions regarding the potential effect of the intervention on ipsilesional M1 excitability.

As a complementary non-invasive measure of intracortical inhibition not well described in children with UCP, we evaluated the CSP duration and how it changed following the intervention. CSP duration is thought to reflect inhibition related to both γ-aminobutyric acid-B (GABA_B_) activity in the motor cortex (Chen et al., 1999; McDonnell et al., 2006), and from inputs to the spinal cord, such as proprioceptive afferents (Inghilleri et al., 1993). Therefore, we cannot rule out that spinal circuits contributed to the CSP duration. Our rationale was that the activity of inhibitory circuits within contralesional M1 may also increase following cathodal tDCS, as demonstrated in a prior study investigating rehabilitation and tDCS in adults with stroke (Goodwill et al., 2016). Using a similar assessment of CSP duration to Rich et al. (2018), we observed increased CSP duration in three of eight individuals in the Active+CIMT group and zero of seven in the Sham+CIMT. Still, the changes in CSP duration were highly variable and were not statistically significant at the group level. Kuo et al. (2018) used paired-pulse TMS paradigms to assess intracortical inhibition in children with UCP, and reported a decrease in the contralesional hemisphere and no change in the ipsilesional hemisphere for short intracortical inhibition following CIMT and repetitive TMS. While information regarding inhibitory mechanisms is helpful in understanding the effects of tDCS interventions, the appropriate and reliable measure for children with UCP remains to be determined.

### Cathodal Contralesional tDCS and Exaggerated IHI

The aim of our combined tDCS/CIMT intervention was to reduce the presumed exaggerated IHI from the contralesional M1 upon the ipsilesional M1. A previous study by Kirton et al. (2010) using repetitive TMS applied to the contralesional hemisphere demonstrated the potential effects of this approach on increasing ipsilesional excitability. Moreover, evidence from typically-developing children indicates that cathodal tDCS applied to the un-trained hemisphere can improve motor skill learning (Ciechanski and Kirton, 2017). Our limited ipsilesional M1 excitability data preclude us from the effects of cathodal tDCS to modulate the ipsilesional hemisphere. However, our previously published behavioral results from this study showed that while all participants saw improvements in hand function, there was no significant effect of Active+CIMT on improvement compared to Sham+CIMT (Gillick et al., 2018b). Similarly, tDCS did not improve hand function in a combined tDCS/motor training study of children with UCP (Kirton et al., 2017). Recent publications have challenged the IHI model, concluding that exaggerated IHI is not ubiquitous in individuals with stroke (Stinear et al., 2015; Boddington and Reynolds, 2017; Bertolucci et al., 2018). Specifically in children with UCP, Eng et al. (2017) showed that IHI was similar between the ipsilesional and contralesional hemispheres. Therefore, decreasing the excitability of the non-lesioned hemisphere using cathodal tDCS may have no effect on ipsilesional excitability (McCambridge et al., 2018). One possible explanation for these results may be related to the timing and location of brain injury in children with UCP. Specifically, studies in adults with subcortical lesions demonstrated exaggerated IHI (Murase et al., 2004), however, this effect may not be applicable in children or adults with cortical lesions. Our sample of children with UCP exhibited both cortical and subcortical lesions, from which we cannot make definitive conclusions regarding the role of IHI. Altogether, these findings question the approach of reducing exaggerated IHI through targeted inhibition of the contralesional hemisphere as a promising approach for future stroke therapy (Eng et al., 2017).

It is worth noting that we detected ipsilateral projections from contralesional M1 to the more-affected FDI which may explain the lack of IHI mechanisms in some individuals. These projections may represent a pattern of brain organization following early brain injury that is associated with poor motor function (Holmström et al., 2010; Mackey et al., 2014) and may be an important indicator of response to interventions (Kuhnke et al., 2008; Smorenburg et al., 2017). We found that when testing contralesional M1, the less-affected FDI MEP amplitude was significantly larger than more-affected FDI MEP amplitude. This result is in agreement with a prior study by Zewdie et al. (2017) describing differences in the neurophysiologic properties of ipsilateral and contralateral projections from the contralesional M1. Characterizing ipsilateral projections is critical because these projections are associated with poorer hand function (Holmström et al., 2010; Smorenburg et al., 2017) and may also undergo neuroplastic changes following movement training (Friel et al., 2016). It is worth noting that MEP amplitudes of ipsilateral (more-affected) projections were measured from the same location as contralateral projections (i.e., the contralesional motor hotspot). Because a new hotspot for ipsilateral responses was not formally assessed in our protocol, doing so may have provided more robust information about the excitability of ipsilateral projections in our sample. Identifying cortical re-organization with TMS allows for an additional understanding of corticospinal plasticity and recovery following injury, and offers a potential target for future combined rehabilitation and neuromodulatory interventions based upon individual characteristics of brain circuitry and excitability.

### Limitations and Future Directions

We recognize the following limitations and additional factors that may be related to our neurophysiologic findings. First, due to lower Pre-test contralesional MEP amplitudes observed in the Sham+CIMT compared to the Active+CIMT group, there may have been a floor effect related to changes in MEP amplitude, even though Pre-test differences in MEP amplitude were adjusted for in the statistical analysis. The overall variability observed in the neurophysiologic measurements may be related to age differences and alterations in the balance of excitation and inhibition related to the on-going development of the nervous system (Hensch and Bilimoria, 2012; Cohen Kadosh et al., 2015). However, we found no significant relationships between MEP amplitude, and CSP duration at Pre-test after adjusting for age. Second, 0.7 mA tDCS may have been too weak to produce measurable changes in corticospinal excitability for all participants. We chose this intensity based on modeling and preliminary safety pilot work, and as limited by regulatory oversight in this initial study. Our preliminary modeling work suggested that an intensity of 0.7 mA produced peak electric fields comparable to those produced by 1.0 mA in an adult (Gillick et al., 2014, 2015a). Indeed, a recent pediatric modeling study indicated that for a given intensity of tDCS, children show higher peak electric field and field spread compared to adults (Ciechanski et al., 2018). Given the variability in peak electric fields induced by tDCS across individuals (Laakso et al., 2015), the intensity of tDCS likely required adjustment based on the participant’s age and brain anatomy (e.g., skull thickness) to produce the optimal intensity for promoting neuroplasticity.

Although no serious adverse events occurred, our results illustrate the challenges of obtaining TMS measures of excitability in children with UCP (Gillick et al., 2016, 2018a). For instance, in five children with high ipsilesional M1 RMTs (range: 71–80), testing was not performed due to the need to use stimulus intensities exceeding the limit of 85% MSO established in our testing protocol. While recent safety reviews indicate that single-pulse TMS is safe in children (Krishnan et al., 2015; Allen et al., 2017), we did not use testing intensities that exceeded 85% of MSO to ensure the comfort and tolerability of participants. Future study protocols using suprathreshold testing intensities up to 100% MSO may allow for a robust assessment of ipsilesional hemisphere neurophysiology, however, this should be weighed against the possibility of the child withdrawing from testing due to discomfort. Continuing to develop strategies to maximize data collection while adhering to best-practices to prioritize safety and tolerability will maximize the number of participants in whom valid measurements can be obtained.

The interaction of tDCS and intensive motor training likely contributed to individual variability in neurophysiologic measures and obscured any potential effects of tDCS (Juenger et al., 2013; Bleyenheuft et al., 2015; Friel et al., 2016). A prior tDCS study in typically-developing adults has shown that combining active movement, as done in our study, can produce paradoxical effects on corticospinal excitability produced by tDCS (Antal et al., 2007). Additional investigation of the immediate (i.e., single-session) effects of tDCS on motor cortex neurophysiology in children with UCP, in the absence of combinatory motor training, will contribute to our understanding of how tDCS influences the developing brain. Based on the complexity of how tDCS impacts brain excitability, individualized approaches are indicated, particularly in participants with brain pathology. Computational modeling of electric fields, as noted above, is one such approach to account for individual differences in brain anatomy, and to estimate how tDCS may influence brain tissue. Validating these models with neurophysiologic and behavioral measurements is a critical need in determining how modeling will be incorporated into future clinical trials of NIBS interventions.

## Conclusion

This proof-of-principle study evaluated the influence of cathodal contralesional tDCS on corticospinal excitability in pediatric participants with UCP. A hypothesized decrease in contralesional excitability was noted in participants in the Active+CIMT group, however, the efficacy of tDCS to modulate corticospinal excitability was not statistically different than the Sham+CIMT group. A more detailed understanding of how tDCS impacts M1 neurophysiology will be essential to inform future clinical trials on the optimal dosing parameters, based on individual brain circuitry, to explore the potential functional benefit of both neuromodulation and motor training.

## Ethics Statement

Before enrolling, participants under the age of 18 provided assent and both caregivers provided written consent; participants over the age of 18 provided written consent. This study was approved by the Institutional Review Board at the University of Minnesota.

## Author Contributions

TR, C-YC, JM, KR, MC, GM, and BG contributed to the design and conception of the study. All authors contributed to the acquisition of data, analysis and interpretation of the results. SN drafted the first version of the manuscript. All authors provided revisions to the manuscript and approved the final version.

## Conflict of Interest Statement

The authors declare that the research was conducted in the absence of any commercial or financial relationships that could be construed as a potential conflict of interest.

## Abbreviations

AHA: Assisting Hand Assessment
CIMT: constraint-induced movement therapy
CSP: cortical silent period
CST: corticospinal tract
EMG: electromyography
FDI: first dorsal interosseous
M1: primary motor cortex
MEP: motor evoked potential
MSO: maximum stimulator output
RMT: resting motor threshold
tDCS: transcranial direct current stimulation
TMS: transcranial magnetic stimulation
UCP: unilateral cerebral palsy.
