# Supplementary material for: Influence of Combined Transcranial Direct Current Stimulation and Motor Training on Corticospinal Excitability in Children With Unilateral Cerebral Palsy
Source: Front Hum Neurosci. 2019 Apr 24;13:137. doi: 10.3389/fnhum.2019.00137 (PMC6492624; doi:10.3389/fnhum.2019.00137)
Supplement: Supplementary file 1 [file Table_1.DOCX]

| **Supplemental Table 1. Individual summary of MEP amplitude values and participants included/excluded from analysis** | | | | | | | | | | | | | | | | | | | | |  |
| --- | --- | --- | --- | --- | --- | --- | --- | --- | --- | --- | --- | --- | --- | --- | --- | --- | --- | --- | --- | --- | --- |
| **Included Participants** | | | | | | | | | | | | | | | | | | | | |  |
|  |  |  |  | | **Ipsilesional Testing** | | | | | | |  | | **Contralesional Testing** | | | | | | |  |
| **Group** |  | **CNS Medications** |  | | **Pre- Test** |  | **Post- Test** | |  | | **FU** | |  | **Pre- Test** | |  | **Post- Test** |  | **FU** | | |
| Sham |  |  |  | | < 3 |  | <3 |  | | Rest | |  | | 547.53 (619.99) | | | 938.82 (358.39) | | 1117 (1057) | |  |
| Sham |  |  |  | | 682.48 (309.82) |  | 644.32 (243.30) | |  | | 460.70 (233.95) | |  | 194.05 (114.11) | | | 465.41 (360.30) | | 300.35 (232.78) | | |
| Sham |  |  |  | | NR |  | NR |  | | NR | |  | | 266.41 (265.03) | | | 498.98 (243.92) | | 474.19 (334.18) | |  |
| Sham |  |  |  | | NR |  | NR |  | | NR | |  | | 959.75 (692.56) | | | 210.88 (137.37) | | 543.44 (325.80) | |  |
| Sham |  |  |  | | NR |  | NR |  | | NR | |  | | 346.92 (260.74) | | | 2240 (1330) | | 1289 (791.8) | |  |
| Active |  | Levetiracetam |  | | 104.00 (26.50) |  | 102.58 (25.36) | |  | | 187.49 (85.15) |  | | 544.39 (262.49) | | | 407.58 (245.67) | | 274.46 (138.97) | | |
| Active |  |  |  | | NR |  | Tol |  | | Tol | |  | | 566.74 (429.57) | | | 321.90 (219.74) | | 733.36 (520.02) | |  |
| Active |  |  |  | | NR |  | NR |  | | NR | |  | | 1565 (1015) | | | 733.52 (447.35) | | 453.31 (237.14) | |  |
| Active |  |  |  | | 178.15 (67.92) |  | 222.56 (148.79) | |  | | 656.59 (608.73) |  | | 752.07 (353.02) | | | 721.91 (452.07) | | 1693 (782.6) | |  |
| Active |  |  |  | | NR |  | NR |  | | NR | |  | | 742.31 (223.60) | | | 436.73 (229.82) | | 355.62 (114.19) | |  |
| **Excluded Participants** | | | | | | | | | | | | | | | | | | | | |  |
| **Group** |  | **CNS Medications** |  | | **Pre- Test** |  | **Post- Test** | | | **FU** | |  | | **Pre- Test** | |  | **Post- Test** | | **FU** | |  |
| Sham |  | Melatonin |  | | NT |  | NT |  | | NT | |  | | NT | |  | NT |  | NT | |  |
| Sham |  | Topiramate |  | | NT |  | NT |  | | Tech | |  | | NT | |  | NT |  | Tech | |  |
| Sham |  |  |  | | NR |  | NR |  | | NR | |  | | NT | |  | NT |  | NT | |  |
| Sham* |  |  |  | | 551.44 (316.00) |  | Tech |  | | 423.25 (206.75) | |  | | 2901 (1950) |  | | 5297 (1462) |  | 2606 (979.4) |  |  |
| Sham |  | Carbamazepine, Citalopram | |  | Thr/Tol |  | Thr/Tol |  | | Thr/Tol | |  | | Tol | |  | 1254 (608.5) |  | 1800 (559.3) |  |  |
| Active |  |  |  | | NR |  | NR |  | | NR | |  | | NT | |  | NT |  | NT | |  |
| Active |  |  |  | | Tech |  | NC |  | | NC | |  | | Tech | |  | NC |  | NC | |  |
| Active |  |  |  | | Thr |  | Thr |  | | Thr | |  | | NT | |  | NT |  | NT | |  |
| Active |  | Sertraline |  | | NT |  | NT |  | | NT | |  | | NT | |  | NT |  | NT | |  |
| Active |  |  |  | | NR |  | NR |  | | NR | |  | | Tech | |  | NC |  | NC | |  |
| Data are mean (SD). FU: 6-month follow-up; MEP: Motor evoked potential; *Excluded from all analyses (active motor threshold), < 3: Less than 3 analyzable trials; NR: No MEP response; NC: No Pre-test comparison; NT: Not tested; Rest: Muscle not at rest during testing; Tol: Unable to toleratre full testing; Tech: Technical issues; Thr: Resting motor threshold too high. | | | | | | | | | | | | | | | | | | | | |  |
