# Supplementary material for: Influence of Combined Transcranial Direct Current Stimulation and Motor Training on Corticospinal Excitability in Children With Unilateral Cerebral Palsy
Source: Front Hum Neurosci. 2019 Apr 24;13:137. doi: 10.3389/fnhum.2019.00137 (PMC6492624; doi:10.3389/fnhum.2019.00137)
Supplement: Supplementary file 2 [file Table_2.DOCX]

| **Supplemental Table 2. Individual summary of CSP duration values and participants included/excluded from analysis** | | | | | | | | | | | | | | | | |
| --- | --- | --- | --- | --- | --- | --- | --- | --- | --- | --- | --- | --- | --- | --- | --- | --- |
| **Included Participants** | | | | | | | | | | | | | | | | |
|  |  |  |  | **Ipsilesional Testing** | | | | | |  | **Contralesional Testing** | | | | | |
| **Group** |  | **CNS Medications** |  | **Pre- Test** |  | **Post- Test** |  | | **FU** |  | **Pre- Test** |  | **Post- Test** |  | | **FU** |
| Sham |  | Melatonin |  | < 3 |  | < 3 | |  | < 3 |  | 124.8 (18.3) |  | 125.7 (20.8) |  | | 148.9 (25.8) |
| Sham |  | Topiramate |  | < 3 |  | < 3 | |  | < 3 |  | 121.9 (7.6) |  | 122.4 (8.8) |  | | 64.5 (17.3) |
| Sham |  |  |  | NR |  | NR | |  | NR |  | 72.2 (21.1) |  | 70.4 (22.0) |  | | 48.2 (7.9) |
| Sham |  |  |  | NR |  | NR | |  | NR |  | 188.6 (12.4) |  | 173.3 (23.4) |  | | 191.5 (7.9) |
| Sham |  |  |  | NR |  | NR | |  | NR |  | 109.3 (16.6) |  | 91.0 (18.5) |  | | 95.9 (11.8) |
| Sham |  | Levetiracetam |  | NR |  | NR | |  | NR |  | 139.8 (11.7) |  | 141.5 (18.6) |  | | 102.8 (21.2) |
| Sham |  |  |  | < 3 |  | 187 (31.7) | |  | 119 (32.4) |  | 77.2 (21.1) |  | 75.4 (20.5) |  | | 41.5 (9.5) |
| Active |  |  |  | NR |  | NR | |  | NR |  | 156.2 (15.2) |  | 151.3 (17.4) |  | | 139.0 (11.1) |
| Active |  |  |  | Thr |  | < 3 | |  | < 3 |  | 109.2 (5.2) |  | 117.8 (17.5) |  | | 116.9 (9.4) |
| Active |  | Sertraline |  | 31.7 (5.62) |  | 73.5 (76.8) | |  | 27.6 (5.08) |  | 91.5 (24.0) |  | 92.8 (35.2) |  | | 125.3 (14.2) |
| Active |  |  |  | NR |  | NR | |  | NR |  | 75.5 (31.8) |  | 68.0 (30.7) |  | | 53.5 (14.3) |
| Active |  |  |  | NR |  | Tol | |  | Tol |  | 51.7 (24.1) |  | 84.9 (25.8) |  | | 53.1 (31.0) |
| Active |  |  |  | NR |  | NR | |  | NR |  | 47.7 (7.65) |  | 86.6 (24.0) |  | | 90.0 (22.7) |
| Active |  |  |  | 27.7 (4.05) |  | 31.2 (6.78) | |  | 26.9 (6.84) |  | 102.9 (41.9) |  | 80.4 (39.3) |  | | 119.1 (30.5) |
| Active |  |  |  | NR |  | NR | |  | NR |  | 170.8 (29.9) |  | 163.2 (14.1) | |  | 84.8 (24.3) |
| **Excluded Participants** | | | | | | | | | | | | | | | | |
| **Group** |  | **CNS Medications** |  | **Pre- Test** |  | **Post- Test** |  | | **FU** |  | **Pre- Test** |  | **Post- Test** | |  | **FU** |
| Sham |  |  |  | < 3 |  | < 3 | |  | 95.1 (49.1) |  | < 3 |  | 92.2 (5.64) |  | | 42.4 (17.7) |
| Sham |  |  |  | * |  | * | |  | * |  | * |  | * |  | | * |
| Sham |  | Carbamazepine, Citalopram |  | Thr/Tol |  | Thr/Tol | |  | Thr/Tol |  | Tol |  | 163.7 (15.0) | |  | 148.3 (33.2) |
| Active |  |  |  | Tech |  | 159 (14.3) | |  | 172 (13.3) |  | Tech |  | 63.8 (12.7) |  | | 60.4 (14.2) |
| Active |  |  |  | < 3 |  | < 3 | |  | < 3 |  | 109.9 (22.1) |  | < 3 |  | | 119.0 (17.3) |
| Data are mean (SD). FU: 6-month follow-up; CSP: Cortical silent period; *Excluded from all analyses (active motor threshold), < 3: Less than 3 analyzable trials; NR: No MEP response; Tol: Unable to tolerate full testing; Tech: Technical issues; Thr: Resting motor threshold too high. | | | | | | | | | | | | | | | | |
